# Arginine inhibits the arginine biosynthesis rate-limiting enzyme and leads to the accumulation of intracellular aspartate in *Synechocystis* sp. PCC 6803

**DOI:** 10.1007/s11103-024-01416-1

**Published:** 2024-03-13

**Authors:** Noriaki Katayama, Takashi Osanai

**Affiliations:** School of Agriculture, Meiji University, 1-1-1, Higashimita, Tama-ku, 214-8571 Kawasaki, Kanagawa Japan

**Keywords:** Cyanobacteria, *Synechocystis*, Arginine biosynthesis, Argininosuccinate synthetase

## Abstract

Cyanobacteria are oxygen-evolving photosynthetic prokaryotes that affect the global carbon and nitrogen turnover. *Synechocystis* sp. PCC 6803 (*Synechocystis* 6803) is a model cyanobacterium that has been widely studied and can utilize and uptake various nitrogen sources and amino acids from the outer environment and media. l-arginine is a nitrogen-rich amino acid used as a nitrogen reservoir in *Synechocystis* 6803, and its biosynthesis is strictly regulated by feedback inhibition. Argininosuccinate synthetase (ArgG; EC 6.3.4.5) is the rate-limiting enzyme in arginine biosynthesis and catalyzes the condensation of citrulline and aspartate using ATP to produce argininosuccinate, which is converted to l-arginine and fumarate through argininosuccinate lyase (ArgH). We performed a biochemical analysis of *Synechocystis* 6803 ArgG (*Sy*ArgG) and obtained a *Synechocystis* 6803 mutant overexpressing *Sy*ArgG and ArgH of *Synechocystis* 6803 (*Sy*ArgH). The specific activity of *Sy*ArgG was lower than that of other arginine biosynthesis enzymes and *Sy*ArgG was inhibited by arginine, especially among amino acids and organic acids. Both arginine biosynthesis enzyme-overexpressing strains grew faster than the wild-type *Synechocystis* 6803. Based on previous reports and our results, we suggest that *Sy*ArgG is the rate-limiting enzyme in the arginine biosynthesis pathway in cyanobacteria and that arginine biosynthesis enzymes are similarly regulated by arginine in this cyanobacterium. Our results contribute to elucidating the regulation of arginine biosynthesis during nitrogen metabolism.

**Supplementary Information:**

The online version contains supplementary material available at 10.1007/s11103-024-01416-1.

## Key message

This study revealed the catalytic efficiency and inhibition of cyanobacterial argininosuccinate synthetase by arginine and demonstrated that a strain overexpressing this enzyme grew faster than the wild-type strain.

## Introduction

Cyanobacteria are prokaryotes that can perform oxygen-evolving photosynthesis, and some cyanobacteria are responsible for 25% of the net oceanic productivity (Rae et al. 2013; Flombaum et al. 2013). Cyanobacteria are observed in various habitats, including freshwater, seawater, and terrestrial habitats, and are major primary producers (Hess 2011). Cyanobacteria have a variety of morphologies, being unicellular or filamentous with or without heterocysts, which are cells specialized in nitrogen fixation (Schirrmeister et al. 2013; Esteves-Ferreira et al. 2017). *Synechocystis* sp. PCC 6803 (hereafter *Synechocystis* 6803) is one of the most studied non-nitrogen-fixing unicellular cyanobacteria (Yu et al. 2013). *Synechocystis* 6803 has properties of a model cyanobacterium, such as entire genome sequence information, natural transformation capability, and resistance to cryopreservation (Yu et al. 2013; Kaneko et al. 1996).

**Fig. 1:**
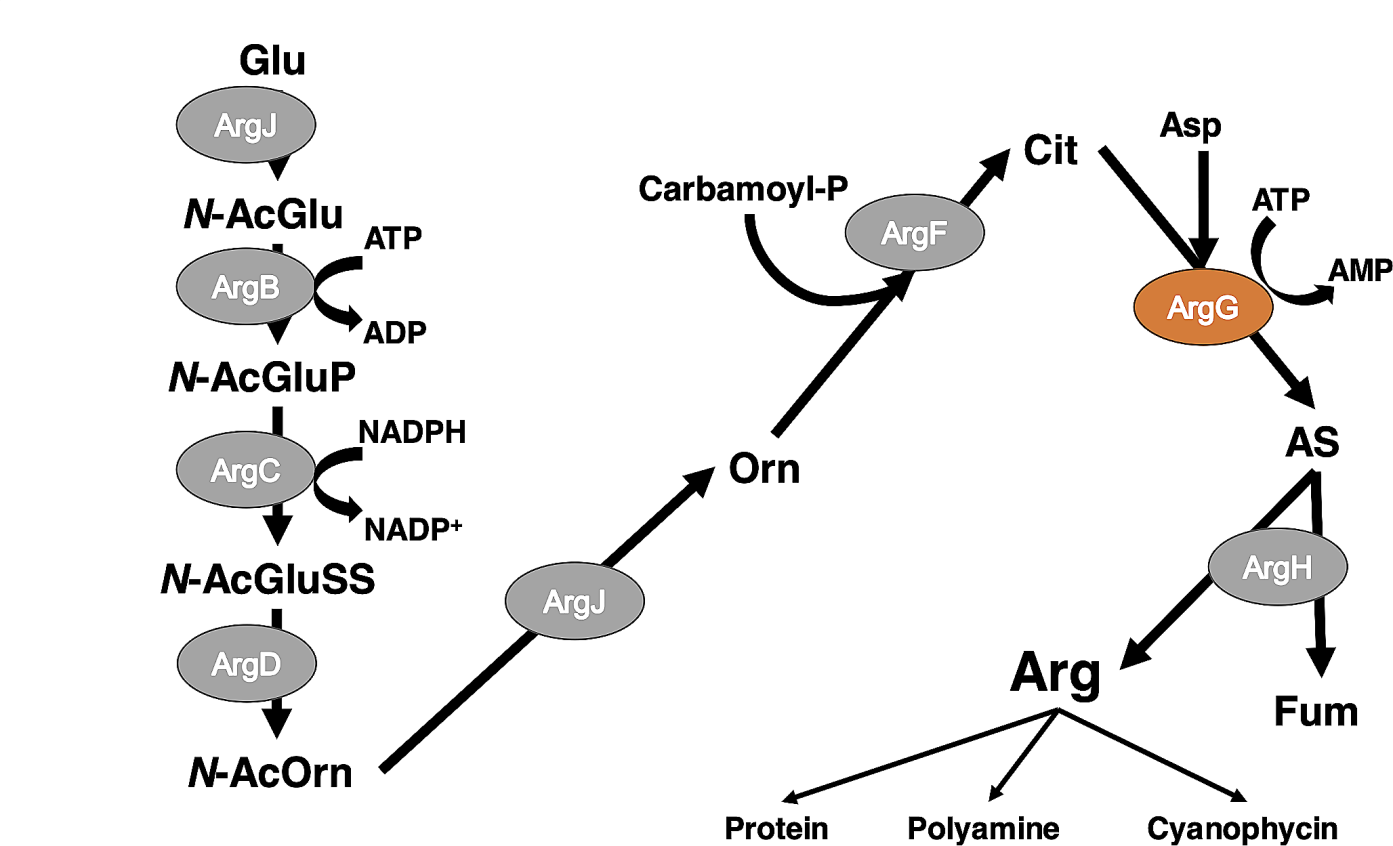
Summary of arginine biosynthesis pathway around reactions catalyzed by ArgG in Synechocystis 6803. This reaction in arginine biosynthesis is based on the previous study (Flores et al. 2019). The gray ellipse represented arginine biosynthesis enzymes. The orange ellipse represented ArgG in this study detailed biochemical analysis was performed. Glu, glutamate; *N*-AcGlu, *N*-acetyl-glutamate; *N*-AcGluP, *N*-acetyl-glutamyl phosphate; *N*-AcGluSS, *N*-acetyl-glutamate semialdehyde; *N*-AcOrn, *N*-acetyl-ornithine; Orn, ornithine; Carbamoyl-P, carbamoyl phosphate; Cit, citrulline; Asp, aspartate; AS, argininosuccinate; Arg, arginine; Fum, fumarate

*Synechocystis* 6803 uses various nitrogen sources such as nitrate (NO_3_^−^), nitrite (NO_2_^−^), ammonium (NH_4_^+^), urea [CO(NH_2_)_2_], and amino acids (arginine, glutamate, and glutamine), preferentially utilizing ammonium (Quintero et al. 2001; Valladares et al. 2002; Flores et al. 2005; Muro-Pastor et al. 2005). *Synechocystis* 6803 grows more rapidly in the presence of ammonium than nitrate (Inabe et al. 2021). After uptake, these inorganic nitrogen sources, except for amino acids, are converted to ammonium and utilized for nitrogen assimilation via the glutamine synthetase-glutamate synthase (GS-GOGAT) cycle (Esteves-Ferreira et al. 2018; Mills et al. 2020). Glutamate is primarily consumed as a nitrogen source and is distributed in various cellular building blocks (Forchhammer and Selim 2020; Inabe et al. 2021).

l-Arginine is synthesized from glutamate through eight enzymatic reactions similar to those in *Escherichia coli* (Fig. 1). *Synechocystis* 6803 has a bifunctional enzyme that catalyzes the first and fifth steps instead of having separate enzymes that catalyze the first and fifth steps (Mills et al. 2020). In cyanobacteria, *N*-acetyl-glutamate kinase (NAGK) is a second-step enzyme that is primarily subject to feedback inhibition by arginine during its biosynthesis (Hoare and Hoare 1966). Cyanobacterial argininosuccinate lyase (ArgH), the last step of arginine biosynthesis, is also inhibited by arginine, and this inhibition for cyanobacterial ArgH changes with pH (Katayama and Osanai 2022). Arginine biosynthesis is also regulated by P_II_ proteins that are widely distributed in bacteria, eukaryotic algae, and plants, and are involved in the regulation of nitrogen assimilation and metabolism (Forchhammer 2004). Arginine accumulates 15-fold compared to the wild-type strain when a mutated P_II_ is introduced strain in *Synechocystis* 6803 (Watzer et al. 2015). Arginine is used as ammonia and glutamate in the catabolizing pathway, and is a precursor for the synthesis of polyamines and cyanobacterial cyanophycin, which is a nitrogen reservoir composed of arginine and aspartate (Flores et al. 2019; Bolay et al. 2021).

Argininosuccinate synthetase (ArgG; EC 6.3.4.5) catalyzes the condensation of citrulline and aspartate into argininosuccinate, the immediate precursor of arginine (Haines et al. 2011). ArgG is considered a rate-limiting step in the urea cycle and nitric oxide (NO) production in mammals (Haines et al. 2011; Jackson et al. 1986; Meijer et al. 1990). The crystal structures of ArgG have been revealed in *E. coli*, *Thermus thermophilus*, and human (Lemke and Howell 2001; Goto et al. 2002, 2003; Karlberg et al. 2008). The kinetic properties of ArgG have been reported mainly in mammals and eukaryotes such as human lymphoblasts, rat liver, and *Saccharomyces cerevisiae* (Kimball and Jacoby 1980; Saheki et al. 1977; Hilger et al. 1979). In *Synechocystis* 6803, the gene encoding argininosuccinate synthetase has been annotated as *argG* (Kaneko et al. 1996). A previous report has suggested that NrrA, a nitrogen-modulated response regulator, regulates ArgG levels for arginine synthesis under nitrogen excess in *Synechocystis* 6803 (Liu and Yang 2014). Argininosuccinate synthetase of *Synechocystis* 6803 from crude cell extracts shows specific activities approximately 3-4-fold lower in the NrrA deletion strain than in the wild-type strain. (Liu and Yang 2014). However, the biochemical properties of *Synechocystis* 6803 ArgG (*Sy*ArgG), such as the regulation by metabolites and catalytic efficiency, have not been reported in detail.

In the present study, we revealed the biochemical characteristics of *Sy*ArgG, which lowered the activity of arginine biosynthetic enzymes and was inhibited by arginine. Physiological essays revealed that *Synechocystis* 6803 accumulated intracellular aspartate when arginine was the sole nitrogen source.

## Material & methods

### Vector construction and expression of recombinant proteins

The genomic region containing the argininosuccinate synthetase open reading frame (ORF) in *Synechocystis* sp. PCC 6803 (*slr0585*) was obtained from the KEGG database (https://www.genome.jp/kegg/). A *Bam*HI-*Xho*I fragment was artificially synthesized by Eurofin Genomics Co. Ltd., and the resultant fragment was cloned into the *Bam*HI-*Xho*I sites of pGEX-6P-1 (GE Healthcare Japan, Tokyo, Japan).

The vector was transformed into *E. coli* BL21(DE3) cells (BioDynamics Laboratory Inc., Tokyo, Japan). Five L of transformed *E. coli* was cultivated in LB medium at 30 °C with shaking (150 rpm), and protein expression was induced overnight in the presence of 0.1 mM isopropyl-β-d-1-thiogalactopyranoside (FUJIFILM Wako Pure Chemical, Osaka, Japan).

### Affinity purification of recombinant proteins

Centrifuged *E. coli* BL21(DE3) cells resuspended in 40 mL PBST (0.137 M NaCl, 2.7 mM KCl, 8.1 mM Na_2_HPO_4_·12H_2_O, 1.47 mM KH_2_PO_4_, and 0.001% Tween 20) were disrupted by sonication (VC-750, EYELA, Tokyo, Japan) 10 times for 20 s at 20% intensity. The insoluble fraction was removed from the disrupted crude extract by centrifugation at 5800 × *g* for 2 min at 4 °C. The supernatant was transferred to a 50 mL tube and washed with 700 μL of Glutathione Sepharose 4B resin (Global Life Sciences Technologies Japan K.K., Tokyo, Japan), and the mixture was gently shaken for 30 min. After centrifugation, the supernatant was removed, and the resin was resuspended in 900 μL of ice-cold PBST. After washing 10 times, the recombinant proteins were eluted five times with 700 μL of GST elution buffer (50 mM Tris-HCl [pH 8.0], 10 mM reduced glutathione). Proteins were concentrated using a Vivaspin 500 MWCO 50,000 device (Sartorius, Germany) and protein concentrations were analyzed using a Pierce BCA Protein Assay Kit (Thermo Scientific, Rockford, IL, USA). SDS-PAGE was performed to evaluate protein purification by staining with Quick Blue Staining Solution (BioDynamics Laboratory Inc., Tokyo, Japan).

### Enzymatic assay for *Sy*ArgG

*Sy*ArgG activity was determined using 250 pmol of enzyme mixed in 1 mL of assay solution [100 mM Tris-HCl (pH 9.0), 2 mM KCl, 5 mM MgCl_2_, 7.5 mM citrulline, 7.5 mM sodium aspartate, 16 mM potassium phosphoenolpyruvate, 1 mM ATP, 0.2 mM NADH, 10 U myokinase, 10 U pyruvate kinase, and 10 U lactate dehydrogenase (Oriental Yeast Co., Ltd., Tokyo, Japan)]. *Sy*ArgG activity was determined based on the formation of AMP coupled with the oxidation of NADH by myokinase, pyruvate kinase, and lactate dehydrogenase (Schuegraf et al. 1960). Absorbance at 340 nm was monitored using a Hitachi U-3310 spectrophotometer (Hitachi High-Tech, Tokyo, Japan). One unit of *Sy*ArgG activity was defined as the consumption of 1 μmol of NADH per minute. *K*_m_ and *V*_max_ were calculated by curve fitting using KaleidaGraph v4.5 software, and *k*_cat_ was calculated using *V*_max_.

### Cyanobacterial strain and culture conditions

The glucose-tolerant *Synechocystis* sp. PCC 6803 strain (hereafter referred to as the GT strain) is isolated by Williams in 1988 (Williams 1988). *Synechocystis* 6803 was cultivated in 70 mL of modified BG-11 medium, consisting of BG-11_0_ liquid medium (Rippka 1988) supplemented with 20 mM HEPES-KOH (pH 7.8) and 5 mM NaNO_3_ or l-arginine. For the incubation, the cultures were bubbled with 1% (v/v) CO_2_ in the air and incubated at 30 °C under continuous white light (50 μmol photons m^− 2^ s^− 1^). For the cultivation of the mutant strains overexpressing *argG* or *argH*, 70 mL of modified BG-11 medium containing 0.7 μg/mL kanamycin was used. The OD_730_ of the cyanobacterial cultures was measured using a Shimadzu UV-2700 UV-vis spectrometer (Shimadzu, Kyoto, Japan). The OD_730_ was set at 0.4 when the cultivation was started.

### Plasmid construction and transformation of ***Synechocystis*** 6803 cells

**Fig. 2:**
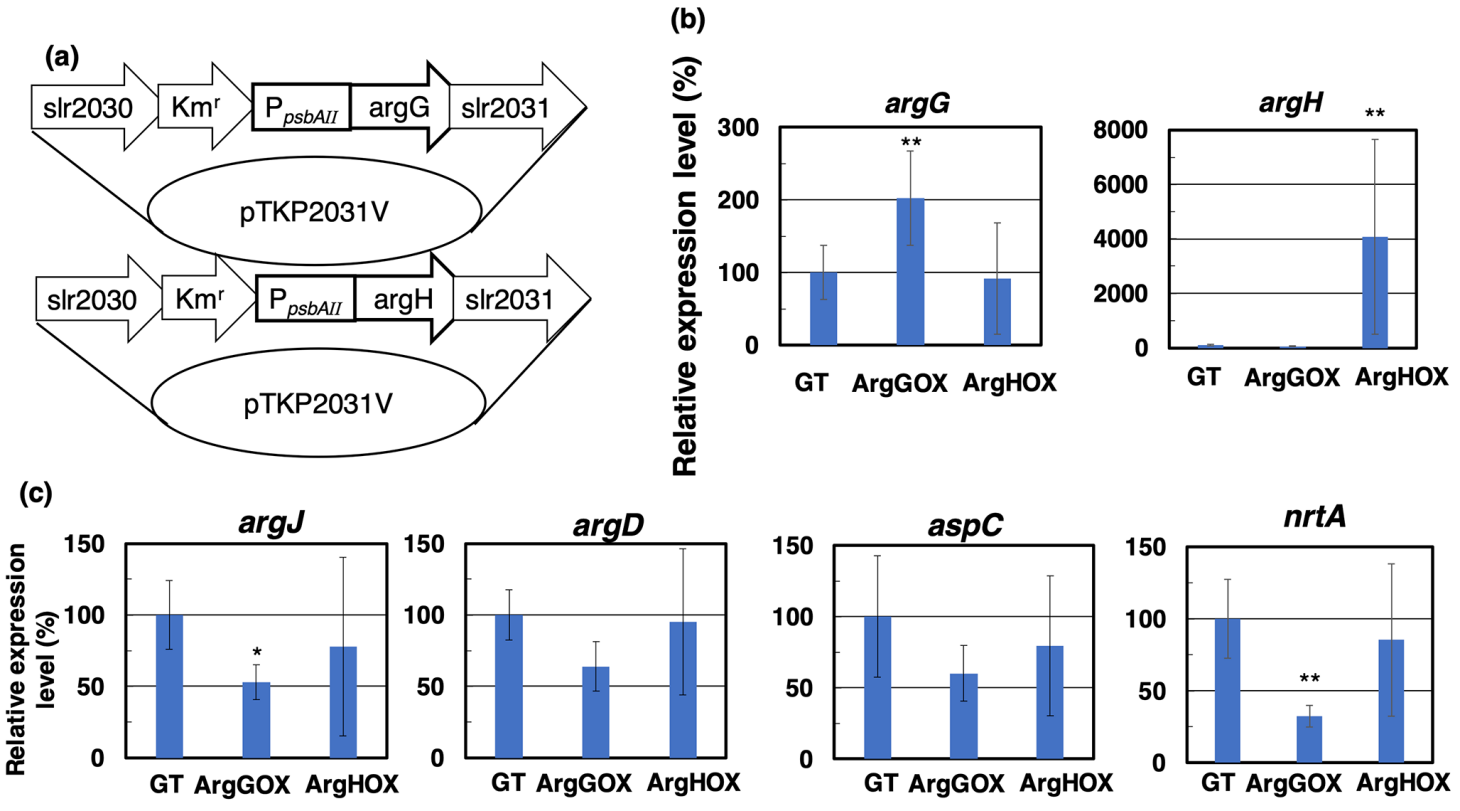
Construction of the ArgGOX and ArgHOX strains. (**a**) Vectors for establishing the ArgGOX and ArgHOX strains. (**b**) The expression levels of *Sy*ArgG and *Sy*ArgH in the GT, ArgGOX, and ArgHOX strains. The data represent the relative amounts of transcript products, and the amount in the GT strain was set at 100%. Data represent the means ± SD obtained from four or five independent experiments. (**c**) The expression levels of arginine biosynthesis-related genes in the GT, ArgGOX, and ArgHOX strains. The data represent the relative amounts of transcript products, and the amount in the GT strain was set at 100%. Data represent the means ± SD obtained from six independent experiments. Statistically significant differences between the activity in the absence or presence of the effector were examined by two-tailed Student’s *t*-test and are represented by asterisks (* = *P* < 0.05, ** = *P* < 0.005)

The open reading frames of *Synechocystis* 6803 *argG* (*slr0585*) and *argH* (*slr1133*) were synthesized and introduced into the *Nde*I-*Eco*RV sites of the pTKP2031 vector containing the *psbAII* promoter by Eurofin Genomics Japan. The *psbAII* promoter and the gene encoding *Sy*ArgG or *Sy*ArgH were integrated into the open reading frames *slr2030* and *slr2031* in *Synechocystis* 6803 genome, along with a kanamycin resistance cassette (Fig. 2a). Transformation of the GT strain was performed by homologous recombination, as described previously (Osanai et al. 2011). The generated strains overexpressing *argG* or *argH* were designated as ArgGOX and ArgHOX, respectively.

### RNA extraction and quantitative real-time PCR

The cyanobacterial cells were harvested for 2 days of photoautotrophic cultivation [OD_730_ × culture volume (mL) = 15] and stored at -30 °C. Frozen cells were suspended in 1 mL ISOGEN (Nippon gene, Tokyo, Japan), gently shaken for 30 min, and then 200 μL of chloroform was added. The mixture was then strongly shaken and centrifuged at 17,900 × *g*, 4 °C, 15 min. Thereafter, 400 μL of isopropanol was added to 400 μL of supernatant and left on ice for 15 min. After centrifugation at 17,900 × *g* for 25 min at 4 °C, 200 μL of ice-cold 70% ethanol was added to the pellet to remove the supernatant. After centrifugation at 17,900 × *g* for 3 min at 4 °C to remove the supernatant, the residue was dried using a centrifugal evaporator CVE-2000 (EYELA, Tokyo, Japan). The pellet containing 20 μg RNA was resuspended and treated with TURBO DNase (Thermo Fisher Scientific, Rockford, IL, USA) for 1 h at 37 °C to remove residual DNA. After the enzymatic reaction, 500 μL of stabilized water and 600 μL of phenol/chloroform/isoamyl alcohol (25:24:1) were added to the solution. The solution was vigorously shaken and centrifuged at 17,900 × *g* for 5 min at 4 °C. Then, 550 μL of supernatant was transferred to another tube, and 55 μL of 3 M sodium acetate and 550 μL of isopropanol were added to the supernatant. The solution was then gently shaken, left for 15 min on ice, and centrifuged at 17,900 × *g* for 25 min at 4 °C. The supernatant was removed and 200 μL of ice-cold 70% ethanol was added to the pellet. After centrifugation at 17,900 × *g* for 3 min at 4 °C to remove the supernatant, the pellet was dried using a centrifugal evaporator CVE-2000. The cDNAs were synthesized using the SuperScriptIII First-Strand Synthesis System (Thermo Fisher Scientific, Rockford IL, USA) from 2 μg of extracted RNA. Quantitative real-time PCR was performed using StepOne Plus (Life Technologies, Carlsbad, CA), and the internal standard was the expression levels of *rnpB* encoding RNase P subunit B, as described previously (Schlebusch and Forchhammer 2010). The base sequence of the using primers for cDNAs amplification are as follows Supplemental Table 1 (Table S1).

### Measurement of the *Sy*ArgG activity from cell extracts

The measurement of enzymatic activity from cell extracts was performed consistent with the previously described method (Ito et al. 2023) and added some modifications. The *Synechocystis* 6803 GT and ArgGOX strains cells were harvested after 3 days of photoautotrophic cultivation (50 μmol photons m^− 2^ s^− 1^) [OD_730_ × culture volume (ml) = 100] with 5 mM NaNO_3_. These cells were resuspended PBS-T and were disrupted by sonication (10 s at 20% intensity) repeated 5 times. After centrifugation (21,400×*g* for 5 min at 4 °C), 900 μL of supernatant was collected, and 40, 200, and 400 μg of total proteins in the supernatant were used for *Sy*ArgG activity assay. The enzymatic reaction catalyzed by *Sy*ArgG was performed in 1 mL of assay solution [100 mM Tris-HCl (pH 7.5 or 9.0), 2 mM KCl, 5 mM MgCl_2_, 7.5 mM citrulline, 7.5 mM sodium aspartate, 16 mM potassium phosphoenolpyruvate, 1 mM ATP, 0.2 mM NADH, 10 U myokinase, 10 U pyruvate kinase, and 10 U lactate dehydrogenase (Oriental Yeast Co., Ltd., Tokyo, Japan)]. The assay solution was incubated at 38 °C for 5 min without sodium aspartate, added to the solution sodium aspartate. *Sy*ArgG activity was determined based on the formation of AMP coupled with the oxidation of NADH by myokinase, pyruvate kinase, and lactate dehydrogenase (Schuegraf et al. 1960). Absorbance at 340 nm was monitored using a Hitachi U-3310 spectrophotometer (Hitachi High-Tech, Tokyo, Japan). As a control, we measured the change of absorbance at 340 nm in the absence of sodium aspartate.

### Extraction and measurement of aspartate and glutamate from ***Synechocystis*** 6803 cells

After 3 days of cultivation, *Synechocystis* 6803 cells [OD_730_ × culture volume (mL) = 100] were harvested by centrifugation [5800 × *g* for 4 min at 25 °C]. The cells were suspended in 600 μL of 60% (v/v) methanol and mixed with TWIN MIXER TM-282 (Asone, Osaka, Japan) for 15 min. The suspension was then centrifuged. A volume of 500 μL of the supernatant was transferred to an Amicon Ultra 3-kDa-cutoff filter (Merck, Billerica, MA, USA) and centrifuged. After centrifugation, 350 μL of the filtered fraction was dried using a CVE-2000 centrifugal evaporator (EYELA, Tokyo, Japan). The pellet was dissolved in 200 μL of 100 mM Tris-HCl buffer (pH 8.0). The quantification of aspartate and glutamate was performed using the Aspartate Assay Kit (MAK095) (Sigma-Aldrich, St. Louis, MO, USA) and F-kit l-glutamate (J.K. International, Tokyo, Japan).

### Extraction cyanophycin and arginine quantification

Cyanophycin contains equal molecules of arginine and aspartate, therefore the concentration of cyanophycin equals the amount of arginine. We performed cyanophycin extraction and arginine quantification using the Sakaguchi reaction according to the studies (Burgstaller et al. 2022, Messineo 1966) with some modifications. After 3 days of cultivation, *Synechocystis* 6803 cells [OD_730_ × culture volume (mL) = 25] were harvested by centrifugation [5800 × *g* for 4 min at 25 °C] and 1 mL acetone was added to the cells and incubation with shaking for 30 min. After centrifugation [13,000 × *g* for 10 min at 25 °C], the supernatant was removed and the residue was resuspended in 1.2 mL of 0.1 M HCl. The samples were incubated and shaken for 60 min at 60. After centrifugation [13,000 × *g* for 10 min at 4 °C], the supernatant was transferred to another 1.5 mL microtube, and added 300 μL of 1 M Tris-HCl (pH9.0) to the supernatant. The sample was incubated on ice for 40 min. After centrifugation [18,000 × *g* for 15 min at 4 °C], the supernatant was removed and resuspended in 1 mL of 0.01 M HCl. The solution was used for the arginine quantification.

Arginine standards with concentrations of 0.06, 0.3 0.6, 1.2, 2.4, 4.8, 6.0 mM in 0.01 M HCl were prepared. 166 μL of reagent A [300 mg potassium iodide in 100 mL distilled water] was added to 166 μL the sample or standard. Subsequently, 500 μL of reagent B [100 mL of 5 M KOH, 2 g potassium sodium tartrate, 0.1 g 2,4-dichloro-1-naphthol, 180 mL absolute ethanol, 0.2 mL NaClO] was added to the sample and was incubated for 60 min at laboratory temperature. After incubation, 166 μL of reagent C [20% (v/v) NaClO with distilled water] was added to the sample and was incubated for 10 min. We measured the absorbance at 520 nm of the sample, using the sample without arginine as a blank.

### Statistical analysis

*P*-values were calculated using a paired two-tailed Student’s *t*-test in Microsoft Excel for Windows (Redmond, WA, USA). All results were obtained from multiple independent experiments (described in detail in individual figure legends).

## Results

### Determination of the kinetics parameters of *Sy*ArgG and its strong inhibition by arginine

**Fig. 3:**
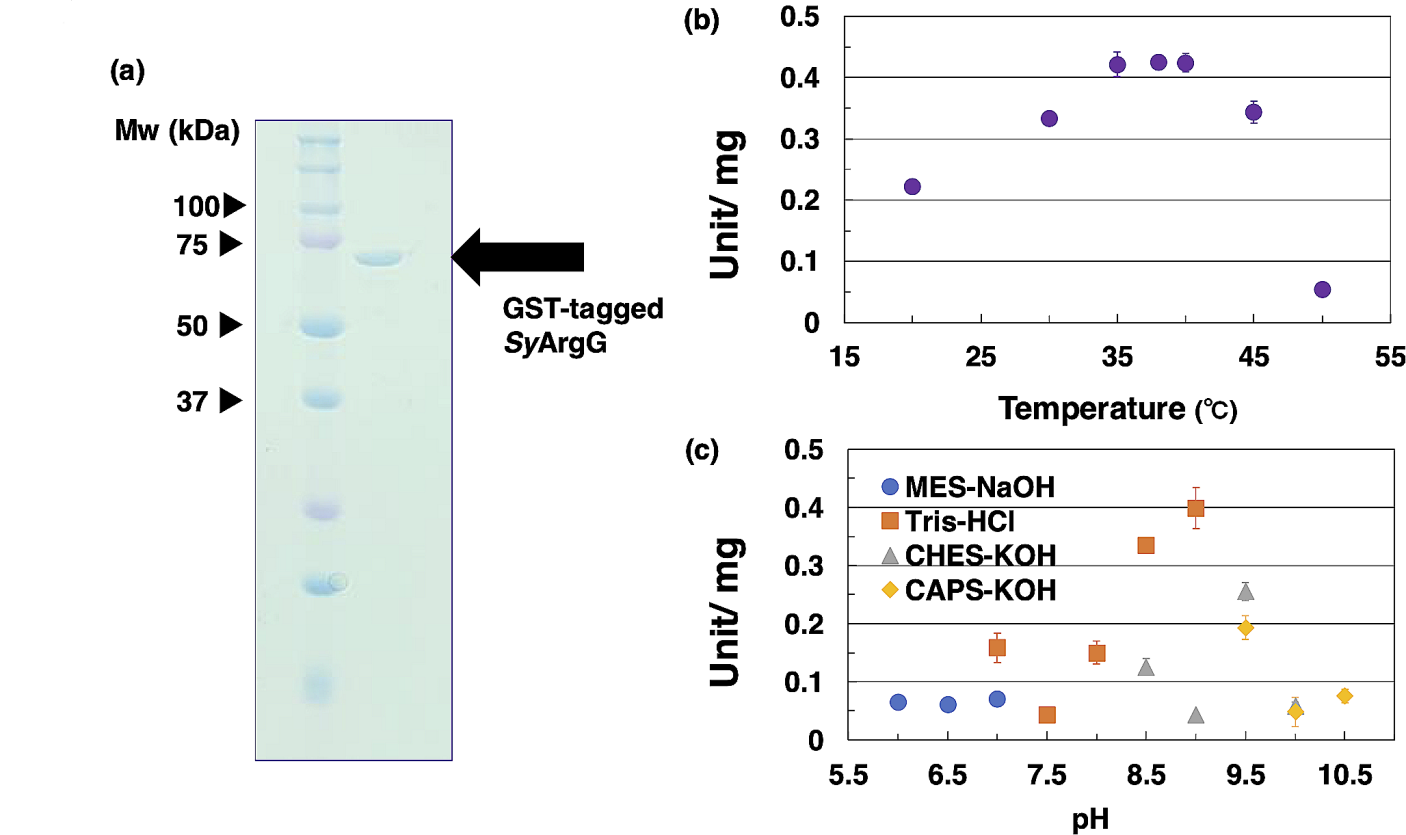
Purification of *Sy*ArgG and determination of its optimal temperature and pH. (**a**) Purified GST-tagged *Sy*ArgG separated on 12% SDS-PAGE gel and stained with Quick Staining Solution. (**b**) Effect of temperature on *Sy*ArgG activity. (**c**) Effect of pH on *Sy*ArgG activity in various buffers. Blue circles, orange squares, gray triangles, and yellow rhombuses represent MES-NaOH, Tris-HCl, CHES-KOH, and CAPS-KOH, respectively

To evaluate the biochemical properties of *Sy*ArgG, glutathione-*S*-transferase-tagged (GST-tagged) *Sy*ArgG was expressed in *E. coli* cells, and GST-tagged *Sy*ArgG was purified from the soluble fraction using affinity chromatography. Purified GST-tagged *Sy*ArgG was confirmed as a single protein band using 12% SDS-PAGE (Fig. 3a). First, we examined the temperature dependence of *Sy*ArgG. The enzyme showed the highest enzymatic activity at 35–40 °C and inactivated above 50 °C (Fig. 3b). We then examined the pH range of *Sy*ArgG activity using various buffers, MES-NaOH, Tris-HCl, CHES-KOH, and CAPS-KOH (Fig. 3c).

**Fig. 4:**
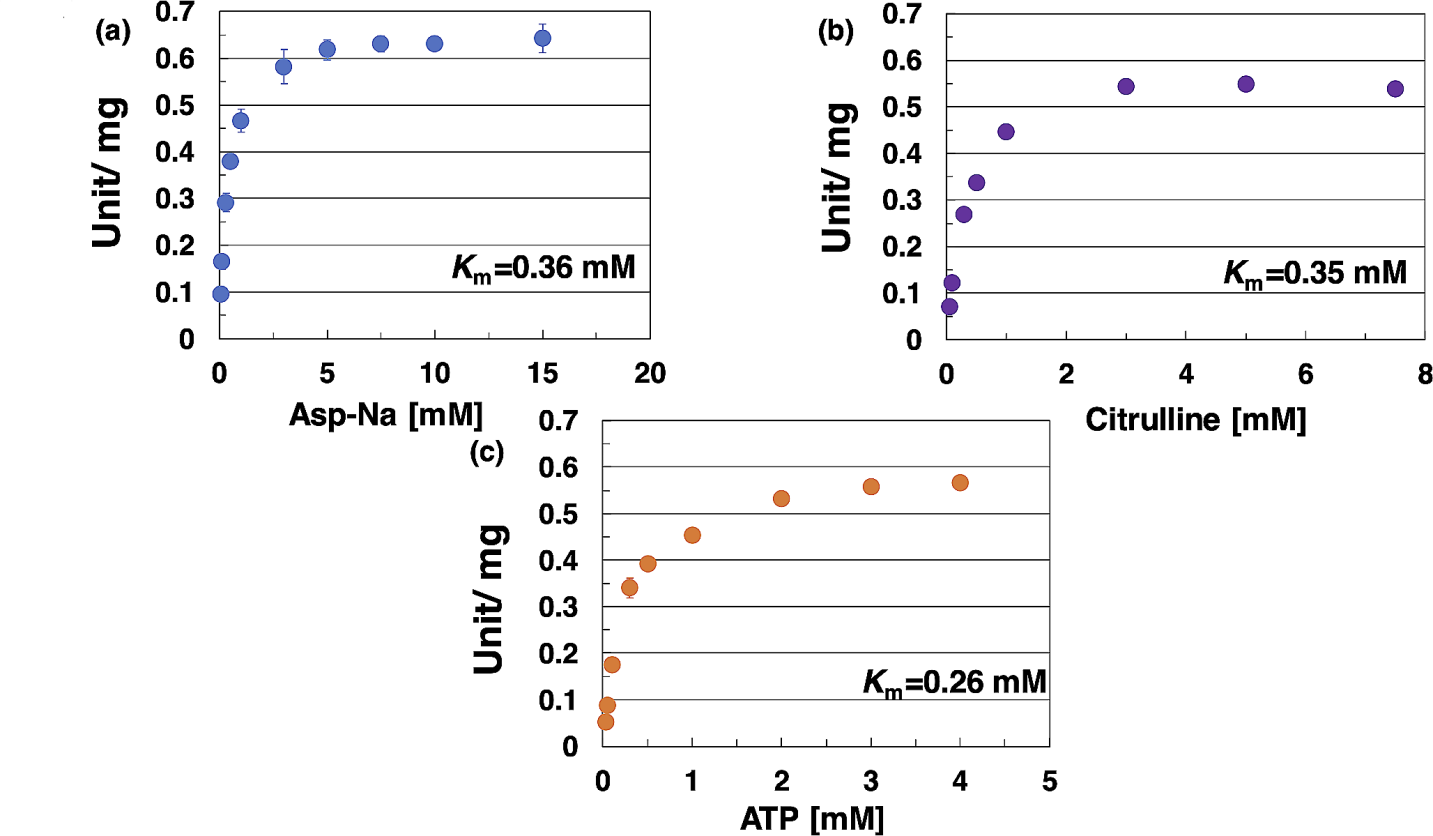
Determination of *K*_m_ values for aspartate, citrulline, and ATP. and the effect of various metabolites on *Sy*ArgG activity. This measurement was performed at 38 °C and pH 9.0. *Sy*ArgG activity was measured by varying (**a**) sodium aspartate, (**b**) citrulline, and (**c**) ATP concentrations. One unit of *Sy*ArgG activity was defined as a consumption of 1 μmol NADH per minute. Data represent the means ± SD obtained from the triplicated independent experiments

Kinetic analyses of *Sy*ArgG based on its saturation curves under 38 °C and pH 9.0 (Fig. 3b and c) for citrulline, aspartate, and ATP, respectively were subsequently performed (Fig. 4a and c). The *K*_m_, *k*_cat_, and *k*_cat_/*K*_m_ values of *Sy*ArgG for citrulline were 0.35 mM, 0.69 s^− 1^, and 1.97 s^− 1^ mM^− 1^, respectively (Table 1). The *K*_m_, *k*_cat_, and *k*_cat_/*K*_m_ values of *Sy*ArgG for aspartate were 0.36 mM, 0.77 s^− 1^, and 2.16 s^− 1^ mM^− 1^, respectively (Table 1). The *K*_m_, *k*_cat_, and *k*_cat_/*K*_m_ values of *Sy*ArgG for ATP were 0.26 mM, 0.7 s^− 1^, and 2.75 s^− 1^ mM^− 1^, respectively (Table 1).

**Table 1:** Kinetic parameters of *Sy*ArgG in optimum condition

Substrate	*K*_m_ (mM)	*k*_cat_ (s^− 1^)	*k*_cat_/*K*_m_ (s^-1^ mM^− 1^ )
Citrulline	0.35 ± 0.01	0.69 ± 0.01	1.97 ± 0.05
Aspartate	0.36 ± 0.04	0.77 ± 0.02	2.16 ± 0.20
ATP	0.26 ± 0.01	0.70 ± 0.002	2.75 ± 0.12

Calculations of the kinetic parameters are described in the Materials and Methods section. Data represent mean ± SD from triplicate independent experiments

**Fig. 5:**
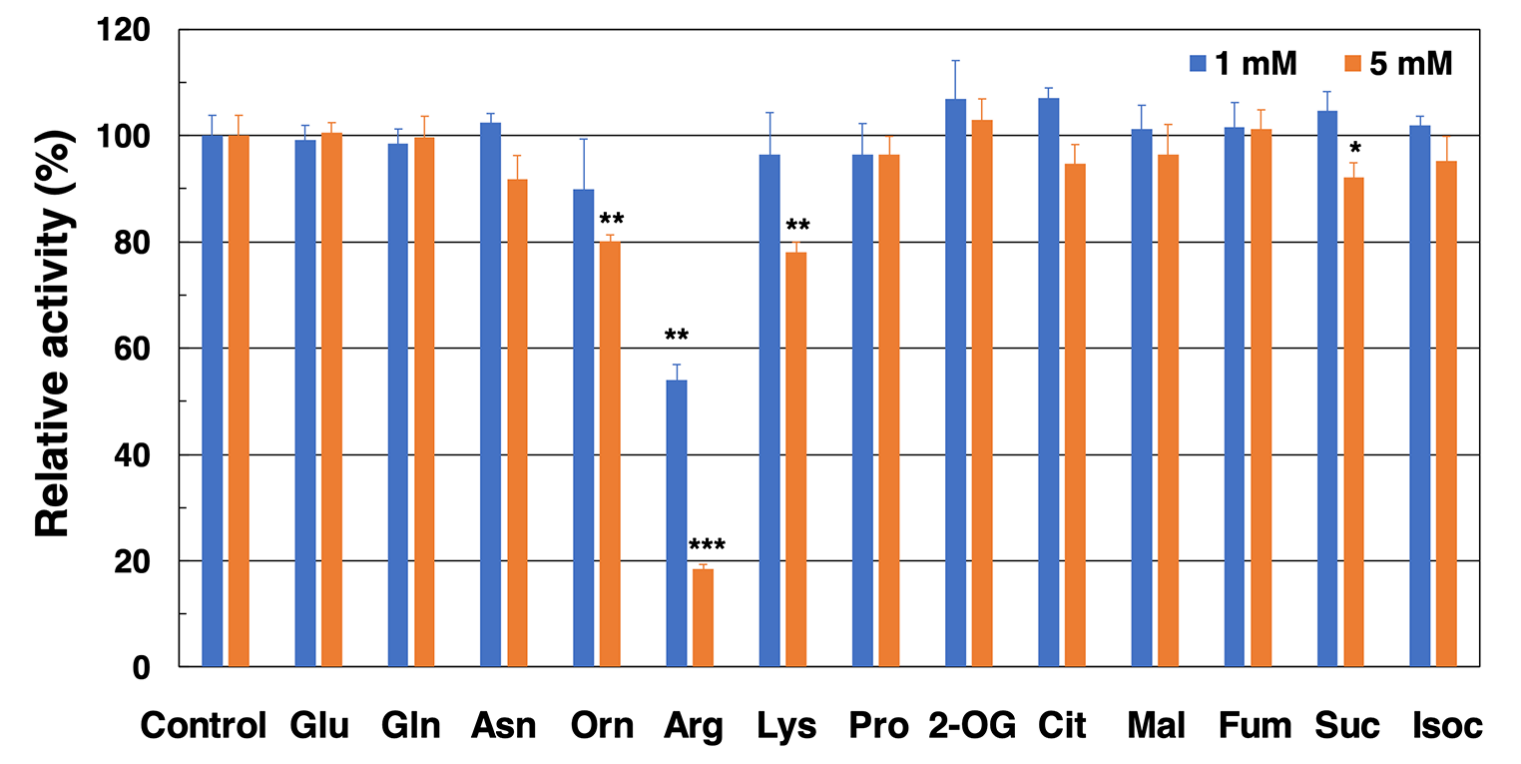
Effect of various metabolites on *Sy*ArgG activity. This measurement was performed at 38 °C and pH 9.0 and fixed concentrations of aspartate, citrulline, and ATP were 7.5, 7.5, and 1.0 mM respectively. *Sy*ArgG activity was represented by relative activity, whereby the activity in the absence of metabolites was set at 100%. Data represent the means ± SD obtained from triplicate independent experiments. Statistically significant differences between the activity in the absence or presence of the effector were examined by two-tailed Student’s *t*-test and are represented by asterisks (* = *P* < 0.05, ** = *P* < 0.005, *** = *P* < 0.0005). Glu, glutamate; Gln, glutamine; Asn, asparagine; Orn, ornithine; Arg, arginine; Lys, lysin; Pro, proline; 2-OG, 2-oxoglutarate; Cit, citrate; Mal, malate; Fum, fumarate; Suc, succinate; Isoc, isocitrate

We measured *Sy*ArgG activity in the presence of several metabolites to determine their effects. *Sy*ArgG activity was measured in the presence of selected organic and amino acids that are closely related to the tricarboxylic acid cycle and arginine biosynthesis (Fig. 5). *Sy*ArgG activity decreased to 53.9% and 18.4% in the presence of 1 mM and 5 mM arginine, respectively (Fig. 5). *Sy*ArgG activity decreased to 80.1, 78.1, and 92.1% in the presence of 5 mM ornithine, lysin, and succinate, respectively (Fig. 5). However, *Sy*ArgG activity did not decrease in the presence of glutamate, glutamine, or asparagine, which have structures similar to that of aspartate (Fig. 5).

### ***Synechocystis*** 6803 cells accumulate aspartate in the medium with arginine

**Fig. 6:**
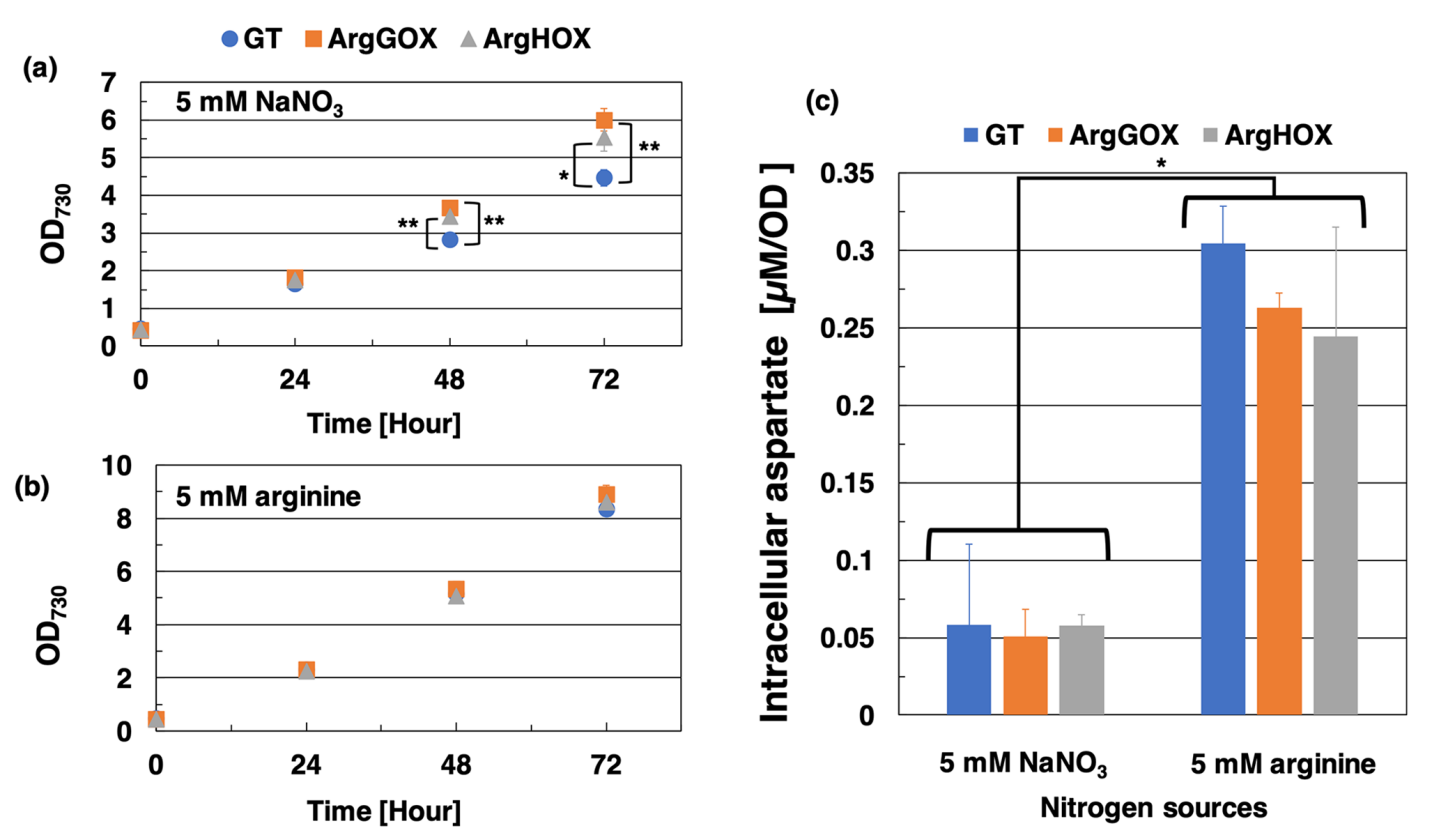
Growth curves of the GT, ArgGOX, and ArgHOX strains under photoautotrophic conditions with (**a**) NaNO_3_ and (**b**) arginine as nitrogen source and intracellular aspartate concentration. The blue circle, orange square, and gray triangle represent the GT, ArgGOX, and ArgHOX strains, respectively. (**c**) Intracellular aspartate concentration in the GT, ArgGOX, and ArgHOX strains with different nitrogen sources. Data represent the means ± SD obtained from triplicate independent experiments. Statistically significant differences between the OD_730_ in the GT, ArgGOX, or ArgHOX strains were examined by paired two-tailed Student’s *t*-tests and are represent by asterisks (* = *P* < 0.05, ** = *P* < 0.005)

To reveal the effects of arginine biosynthesis gene overexpression and different nitrogen sources on growth, we evaluated the GT strains overexpressing the *Sy*ArgG (*slr0585*) and *Sy*ArgH (*slr1133*) genes (designated ArgGOX and ArgHOX) using 5 mM NaNO_3_ as a nitrogen source (Fig. 2a). The RNA expression of the gene encoding *Sy*ArgG in the ArgGOX strain was 2.0-fold higher than that in the GT strain (Fig. 2b). We tried to measure the *Sy*ArgG activity from cell extracts of GT and ArgGOX strains, but the enzymatic activity was not detected, because of low activity of *Sy*ArgG. The RNA expression level of the gene encoding *Sy*ArgH in the ArgHOX strain was 40.8-fold higher than that in the GT strain (Fig. 2b). And we measured RNA expression of the arginine biosynthesis-related genes, such as the *nrtA* encoding nitrate transporter subunit, the *aspC* encoding aspartate aminotransferase, the *argJ* encoding the bifunctional enzyme of arginine biosynthesis, the *argD* encoding the bifunctional aminotransferase catalyzed arginine biosynthesis and GABA metabolism. The expression levels of the gene encoding *argJ (sll1883)* and *nrtA (sll1450)* in the ArgGOX strain were 0.53- and 0.32-fold lower than those in the GT strain, respectively (Fig. 2c). The expression levels of the gene encoding *argD (slr1022)* and *aspC (sll0402)* were not significantly different among the GT, ArgGOX, and ArgHOX strains (Fig. 2c). We also measured RNA expression of the *argB* gene encoding NAGK that is a significant regulator subjected to arginine feedback inhibition. The expression level of the *argB* encoding NAGK in the ArgGOX strain was 0.37-fold lower than in the GT strain (Fig. S1). The growth of the ArgGOX and ArgHOX strains was faster than that of the GT strain under photoautotrophic conditions with 5 mM NaNO_3_ as a nitrogen source (Fig. 6a). The cell densities of ArgGOX and ArgHOX strains at 72 h were 34% and 24% increase compared to GT strain, respectively (Fig. 6a). In contrast, the growth of these strains was similar under photoautotrophic conditions with 5 mM arginine as a nitrogen source (Fig. 6b). The RNA expression level of the gene encoding *Sy*ArgG in the ArgGOX strain and the gene encoding *Sy*ArgH in the ArgHOX strain was 2.1-fold and 49.6-fold higher than those in the GT strain using as a 5 mM arginine (Fig. S1). The RNA expression levels of other arginine biosynthesis-related genes showed no significant change between the GT strain and ArgGOX or ArgHOX strains (Fig. S2).

Intracellular aspartate levels in the GT, ArgGOX, and ArgHOX strains under photoautotrophic conditions with 5 mM NaNO_3_ or arginine as the nitrogen source was then determined (Fig. 6c). The intracellular aspartate concentration with 5 mM NaNO_3_ was 0.06, 0.05, and 0.06 μM/OD in the GT, ArgGOX, and ArgHOX strains, respectively (Fig. 6c). The intracellular aspartate concentration with 5 mM arginine were 0.30, 0.26, and 0.24 μM/OD in the GT, ArgGOX, and ArgHOX strains, respectively (Fig. 6c). The aspartate concentration in each strain with 5 mM arginine was 5.21, 5.18, and 4.21-fold higher than that in the strains with 5 mM NaNO_3_ (Fig. 6c). We determined intracellular arginine from purified cyanophycin under photoautotrophic conditions with 5 mM NaNO_3_ or arginine as nitrogen source. The intracellular arginine of GT and ArgGOX, ArgHOX strains were 0.27 and 0.26, 0.26 mM, respectively in 5 mM NaNO_3_ (Fig. S3). Intracellular arginine of GT and ArgGOX, ArgHOX strains were 0.80 and 0.88, 0.88 mM, respectively in 5 mM arginine (Fig. S3). We determined intracellular glutamate under photoautotrophic conditions with 5 mM NaNO_3_ or arginine as a nitrogen source. The intracellular glutamate of GT, ArgGOX, and ArgHOX strains were 2.56, 1.85, and 3.24 mM, respectively in 5 mM NaNO_3_ (Fig. S4). Intracellular glutamate of GT, ArgGOX, and ArgHOX strains were 2.44, 2.89, and 3.98 mM, respectively in 5 mM arginine (Fig. S4).

## Discussion

In this study, we revealed the biochemical properties of *Sy*ArgG, a rate-limiting enzyme in the arginine biosynthesis pathway, and that the ArgGOX and ArgHOX strains had different cell densities compared to those of the GT strain.

The *K*_m_ values of *Sy*ArgG for citrulline and aspartate were similar (Table 1). The *K*_m_ values of argininosuccinate synthetase of the higher plant *Pinus pinaster* Ait. (*Pp*ASSY) are both 0.12 mM for citrulline and aspartate (Urbano-Gámez et al. 2020). In mammals, the *K*_m_ values of argininosuccinate synthetase for citrulline and aspartate are 0.02 and 0,078 in rats and 0.044 and 0.068 mM in humans, respectively (Saheki et al. 1977; Kimball and Jacoby 1980). Compared with these reports, our results showed that the *K*_m_ value of cyanobacterial argininosuccinate synthetase was higher than that of the eukaryotic argininosuccinate synthetase. This suggests that *Synechocystis* 6803 can synthesize a precursor of arginine only under conditions of sufficient nitrogen concentration because of the low substrate affinity of *Sy*ArgG. The specific activity of *Sy*ArgG towards the substrates was lower than 1 U/mg (Fig. 4). The specific activity of the key enzyme in arginine biosynthesis, NAGK, is higher than 3 U/mg in the absence of arginine in *Synechocystis* 6803 (Bolay et al. 2021). In addition, the activity of the arginine biosynthesis key enzyme of *Synechocystis* 6803, *N*-acetylornithine aminotransferase, for *N*-acetylornithine (*k*_cat_/*K*_m_: 19.3 s^− 1^ mM^− 1^) was approximately 10-fold higher than that of *Sy*ArgG (*k*_cat_/*K*_m_: 1.97 s^− 1^mM^− 1^) (Li et al. 2023; Table 1). *Synechocystis* 6803 cell crude extract experiments also show that *N*-acetylornithine aminotransferase activity is 2-fold higher than argininosuccinate synthetase activity (Liu and Yang 2014). The *k*_cat_/*K*_m_ of *Sy*ArgG for the substrate (citrulline: 1.97 s^− 1^mM^− 1^, aspartate: 2.16 s^− 1^mM^− 1^) was lower than that of the last-step enzyme in arginine biosynthesis (*Sy*ArgH: 37.3 s^− 1^mM^− 1^) (Katayama and Osanai 2022). These results indicate that *Sy*ArgG is the rate-limiting enzyme in arginine biosynthesis.

*Sy*ArgG enzymatic activity was significantly inhibited by arginine in a concentration-dependent manner (Fig. 5). Argininosuccinate synthetase activities of the thermophilic eubacteria and archaeobacteria *Thermus aquaticus*, *Thermotoga maritima*, and *Sulfolobus solfataricus* are also inhibited by 90% by 8-, 4-, and 2-mM arginine, respectively (Van de Casteele et al. 1990). *Pp*ASSY activity is significantly inhibited by 10 mM arginine by approximately 20%, and *Pp*ASSY activity is similarly inhibited by arginine concentrations ranging from 1 mM to above 20 mM (Urbano-Gámez et al. 2020). These results indicate that *Sy*ArgG is more sensitively regulated by feedback inhibition than *Pp*ASSY. This arginine inhibition is similar to that of previously reported arginine biosynthesis enzymes (NAGK and *Sy*ArgH) in cyanobacteria (Hoare and Hoare 1966; Bolay et al. 2021; Katayama and Osanai 2022). Under physiological conditions, the intracellular arginine concentration in *Synechocystis* 6803 is 0.6 mM (Zhang et al. 2018). The intracellular arginine levels in *Synechocystis* 6803 change depending on the growth medium (Iijima et al. 2020). We propose that arginine biosynthesis in *Synechocystis* 6803 is regulated by *Sy*ArgG, including the ornithine cycle, and not only by NAGK to adapt to various environments.

Under photoautotrophic conditions, ArgGOX and ArgHOX grew faster than the GT strain with 5 mM NaNO_3_ as a nitrogen source (Fig. 6a). This phenotype indicates that overexpression of arginine biosynthesis genes relieves the enzymatic rate-limiting step using NaNO_3_ as a nitrogen source. The mutated P_II_ protein introduced strain shows a similar cell density to the wild-type strain with nitrate as a nitrogen source (Watzer et al. 2015). The *nrrA* deletion mutant has a similar growth rate during the exponential phase, but a longer lag phase leading up to the exponential phase (Liu and Yang 2014). When 5 mM NaNO_3_ was used as the nitrogen source, direct overexpression of arginine biosynthesis genes showed an increase in cell density (Fig. 6a). The *argJ*, *nrtA*, and *argB* expression levels were decreased in the ArgGOX strain compared to the GT strain with 5 mM NaNO_3_ condition (Fig. 2 and S1). The *argB* encoding NAGK that enzyme reaction uses ATP, similar to *nrtA* encoding nitrate transporter subunit. *Sy*ArgG also used ATP for the condensation of citrulline and aspartate (Fig. 4). The *argJ* encoded ArgJ, the enzyme introduces glutamate into arginine biosynthesis and ornithine into the ornithine cycle. We suggested that arginine biosynthesis may regulate to balance of ATP consumption. We suggested that *argG* overexpression controlled the introduction of new substrates into arginine biosynthesis and reduced unnecessary ATP consumption. In contrast, the cell densities of the three strains were similar when 5 mM arginine was used as the nitrogen source (Fig. 6b). *Synechocystis* 6803 produces and accumulates cyanophycin, resulting in increased intracellular arginine levels in BG-11 medium supplemented with 5 mM arginine and 10 mM glucose (Burgstaller et al. 2022). In this study, the intracellular arginine of these strains with 5 mM arginine was higher than those with 5 mM NaNO_3_ (Fig. S3). *Synechocystis* 6803 can uptake arginine from the media, and its uptake activity is 31-fold higher than that of the nitrogen-fixing heterocystous cyanobacteria *Nostoc* (*Anabaena*) sp. PCC 7120 (Montesinos et al. 1997). The majority of arginine taken up from the medium is incorporated into cyanophycin (Stephan et al. 2000). These results suggest that *Synechocystis* 6803 uptakes arginine from the medium and arginine is incorporated into cyanophycin, and intracellular arginine inhibits *Sy*ArgG and other arginine biosynthesis enzymes, resulting in attenuation of the overexpression effects. Furthermore, intracellular aspartate accumulated in all strains when 5 mM arginine was used as the nitrogen source (Fig. 6c). But intracellular glutamate decreased in the ArgGOX strain with 5 mM NaNO_3_ and increased with 5 mM arginine (Fig. 4S). The transcript levels of glutamine synthetase were increased, while transcript levels of glutamine synthetase-inactivating factor IF7 (*gifA*) and IF17 (*gifB*) with arginine-grown WT strain versus nitrate-grown WT strain (Schriek et al. 2008). The ArgGOX strain with 5 mM NaNO_3_ occurs a similar phenomenon, consequently, the intracellular glutamate of the ArgGOX strain was decreased. The amino acid profile of *Synechocystis* 6803 in artificial seawater showed that the levels of ornithine and aspartate were higher than those in cells grown in BG-11 medium (Iijima et al. 2015). *Sy*ArgG activity decreased by 80% in the presence of 5 mM ornithine (Fig. 5). We suggest that arginine biosynthesis enzymes, including *Sy*ArgG, are inhibited by intracellular arginine and that the precursor aspartate is not used as a substrate for *Sy*ArgG and accumulates in *Synechocystis* 6803 cells. We may hypothesize arginine biosynthesis gene overexpression and using arginine as a nitrogen source affects other amino acid metabolism.

In this study, we demonstrated that *Sy*ArgG is the rate-limiting step in arginine biosynthesis in *Synechocystis* 6803. This study contributes to the elucidation of nitrogen metabolism, regulation of arginine biosynthesis, and not only NAGK regulation in *Synechocystis* 6803. Under photoautotrophic conditions, the ArgGOX and ArgHOX strains grew faster than the GT strain. Optimized nitrogen and phosphorus supply yielded a higher productivity of glycogen and PHB (poly-3-hydroxybutyrate) and had little negative effect on biomass production (Singhon et al. 2021). These arginine biosynthesis-overexpressing strains will increase biomass and polyamine productivity in *Synechocystis* 6803.

### Electronic supplementary material

Below is the link to the electronic supplementary material.

Supplementary Material 1

Supplementary Material 2

Supplementary Material 3

Supplementary Material 4

Supplementary Material 5

## Data Availability

Not applicable.
