# Supplementary material for: Arginine inhibits the arginine biosynthesis rate-limiting enzyme and leads to the accumulation of intracellular aspartate in Synechocystis sp. PCC 6803
Source: Plant Mol Biol. 2024 Mar 13;114(2):27. doi: 10.1007/s11103-024-01416-1 (PMC10937788; doi:10.1007/s11103-024-01416-1)
Supplement: Supplementary file 1 — Supplementary Material 1 [file 11103_2024_1416_MOESM1_ESM.docx]

**
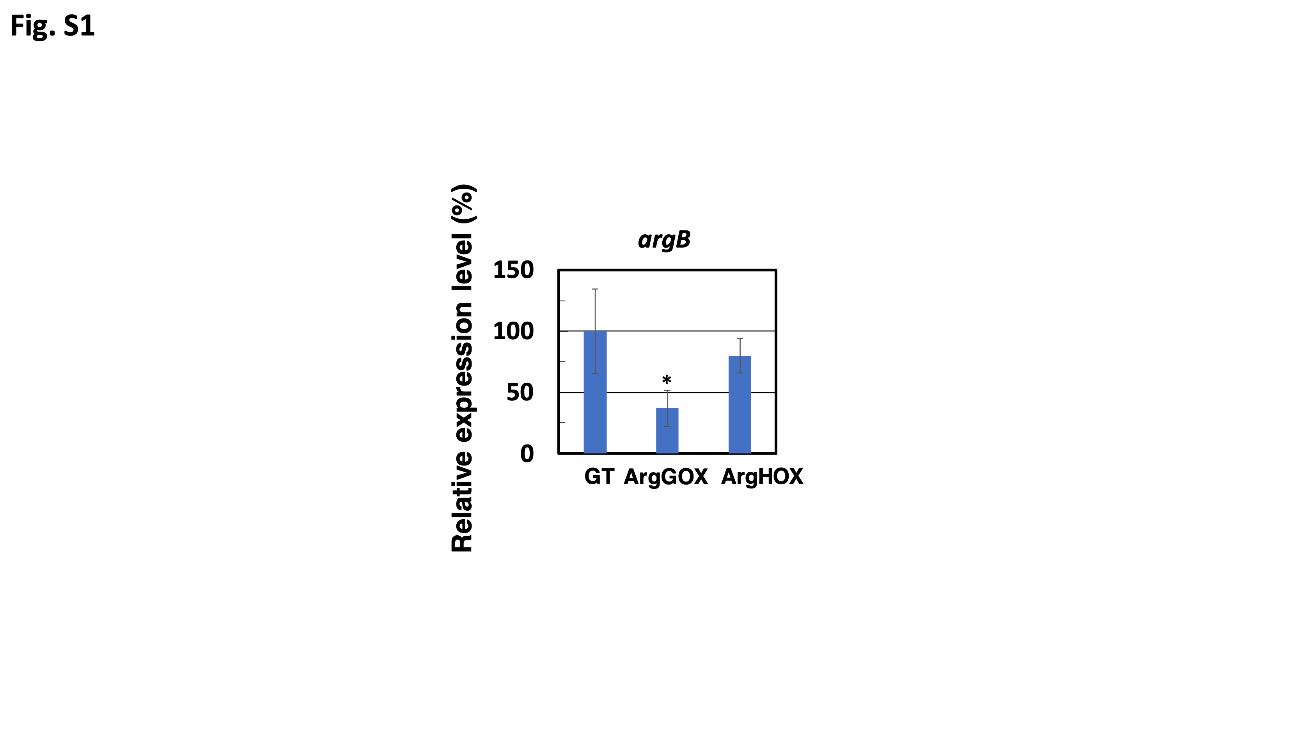
**

**Figure S1** The RNA expression level of the *argB* gene in the GT, ArgGOX, and ArgHOX strains with 5 mM NaNO_3_ as a nitrogen source. The data represent the relative amounts of transcript products, and the amount in the GT strain was set at 100%. Data represent the means ± SD obtained from four or five independent experiments. Statistically significant differences between the activity in the absence or presence of the effector were examined by two-tailed Student’s *t*-test and are represented by asterisks (* = *P* < 0.05,
