# Supplementary material for: Arginine inhibits the arginine biosynthesis rate-limiting enzyme and leads to the accumulation of intracellular aspartate in Synechocystis sp. PCC 6803
Source: Plant Mol Biol. 2024 Mar 13;114(2):27. doi: 10.1007/s11103-024-01416-1 (PMC10937788; doi:10.1007/s11103-024-01416-1)
Supplement: Supplementary file 3 — Supplementary Material 3 [file 11103_2024_1416_MOESM3_ESM.docx]

**
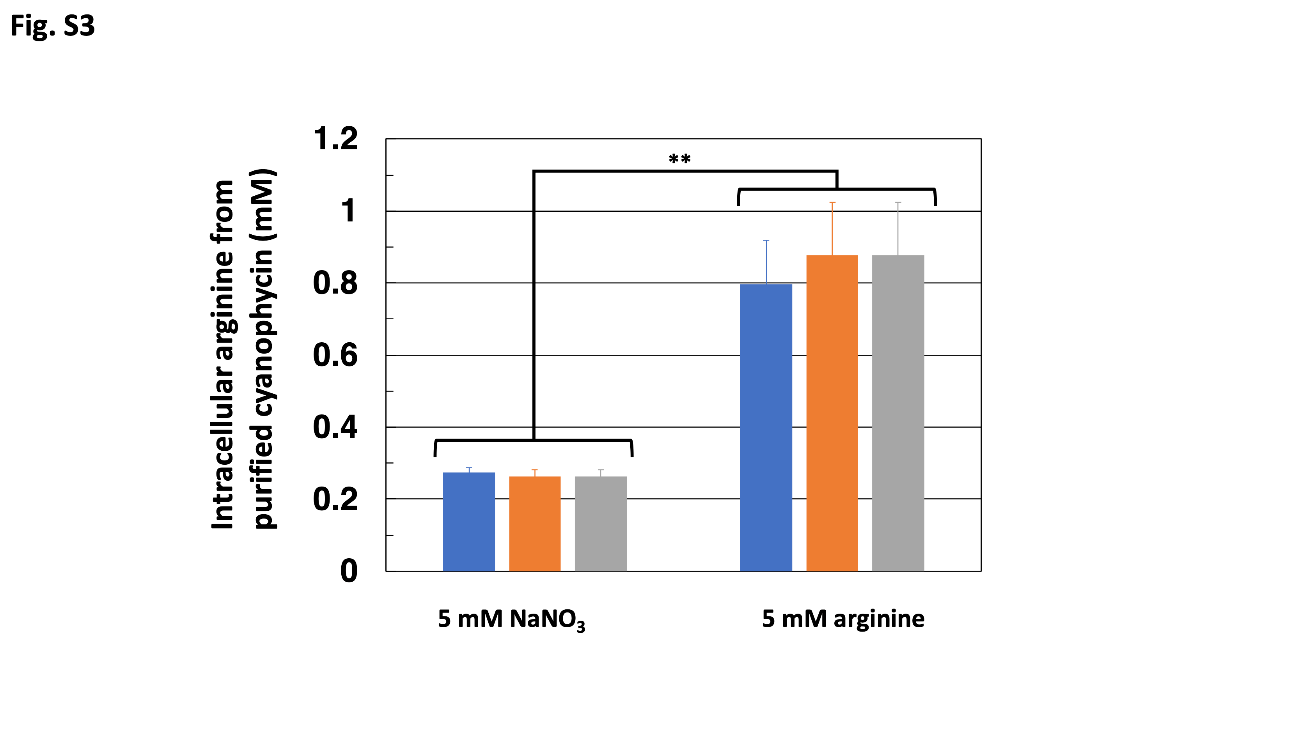
**

**Figure S3** Intracellular arginine concentration from purified cyanophycin in the GT, ArgGOX, and ArgHOX strains with different nitrogen sources. Data represent the means ± SD obtained from triplicate independent experiments. Statistically significant differences between those strains with 5 mM NaNO_3_ and with 5 mM arginine were examined by paired two-tailed Student's *t*-tests and are represent by asterisks (** = *P* < 0.005)
